# FSC-Q: a CryoEM map-to-atomic model quality validation based on the local Fourier shell correlation

**DOI:** 10.1038/s41467-020-20295-w

**Published:** 2021-01-04

**Authors:** Erney Ramírez-Aportela, David Maluenda, Yunior C. Fonseca, Pablo Conesa, Roberto Marabini, J. Bernard Heymann, Jose Maria Carazo, Carlos Oscar S. Sorzano

**Affiliations:** 1 Biocomputing Unit, National Center for Biotechnology (CSIC), Darwin 3, Campus Univ. Autónoma de Madrid, Cantoblanco, 28049 Madrid, Spain; 2 Univ. Autónoma de Madrid, Campus Univ. Autónoma de Madrid, Cantoblanco, 28049 Madrid, Spain; 3 Laboratory of Structural Biology Research, NIAMS, NIH, Bethesda, MD USA; 4 Univ. CEU San Pablo, Campus Urb. Montepríncipe, Boadilla del Monte, 28668 Madrid, Spain

**Keywords:** Electron microscopy, Cryoelectron microscopy

## Abstract

In recent years, advances in cryoEM have dramatically increased the resolution of reconstructions and, with it, the number of solved atomic models. It is widely accepted that the quality of cryoEM maps varies locally; therefore, the evaluation of the maps-derived structural models must be done locally as well. In this article, a method for the local analysis of the map-to-model fit is presented. The algorithm uses a comparison of two local resolution maps. The first is the local FSC (Fourier shell correlation) between the full map and the model, while the second is calculated between the half maps normally used in typical single particle analysis workflows. We call the quality measure “FSC-Q”, and it is a quantitative estimation of how much of the model is supported by the signal content of the map. Furthermore, we show that FSC-Q may be helpful to detect overfitting. It can be used to complement other methods, such as the Q-score method that estimates the resolvability of atoms.

## Abstract (web summary)

Here, the authors present a cryo-EM validation method for the local analysis of the map-to-model fit and introduce FSC-Q as a quality measure. FSC-Q provides a quantitative estimation of how much of the model is supported by the signal content of the map.

## Introduction

Single-particle cryo-electron microscopy (cryoEM) has become a powerful technique for the structural determination of biological macromolecules. In recent years, new direct detection cameras and better reconstruction algorithms yielded maps with high levels of detail. The next logical step is to build an atomic model that fits into the density map. Both, the quality of the reconstructed map and the derived model must be evaluated carefully to identify errors and poorly resolved regions. The quality of the maps is usually expressed in terms of a resolution measure. The typical resolution measure reported is based on a threshold in the Fourier shell correlation (FSC) curve between two independent reconstructions^1^. The agreement of a model with a map can be done in a similar way by calculating the FSC between the experimental map and a map calculated from the model.

However, it is well known that the quality of a reconstruction depends on the local regions of the macromolecule and the resolution must therefore be calculated locally^2–5^, and even directionally^6,7^ (although we will not consider directional effects in this work). To evaluate the quality of an atomic model, different steric measurements have been integrated into tools, such as Molprobity^8^. However, these measures are based on the model itself, regardless of the cryoEM map. Local map-to-model fit measurements have been introduced, such as EMRinger^9^, which takes into account density values near carbon-ß atom, and the recent *Q*-score^10^, which measures the correlation between the map values at points around the atom and a reference Gaussian-like function.

In this paper, we present a measure (FSC-*Q*) for local quality estimation of the fit of the atomic model to the Coulomb potential map. The key difference with previously proposed methods is that we objectively and quantitatively estimate the specific parts of the model that are truly supported by the map on the basis of its local resolution. The rationale of the method is based on the differences between local resolution values calculated with blocres^2^, providing local estimations of FSC values. The process involves the subtraction of the local resolution map between the final full map and the map generated from the atomic model, from the local resolution map between the two half maps (where a half-map refers to a map reconstructed from half of the data set). This latter subtraction, when properly statistically scaled, provides an estimation of the signal content of the map itself, that is then compared with the signal content implied in the fitting of the structural model. When the fitting would require a signal content that, simply, is not present in the experimental data, FSC-*Q* flags it, providing an objective assessment on how the structural model is supported, revealing potential modeling errors, as well as poorly defined parts of models. The approach is conceptually simple, fully objective, and quantitative, and lends itself very well to decision-making in a meta (or multi) criteria approach^11^.

## Results

### Fitting analysis

**Fig. 1:**
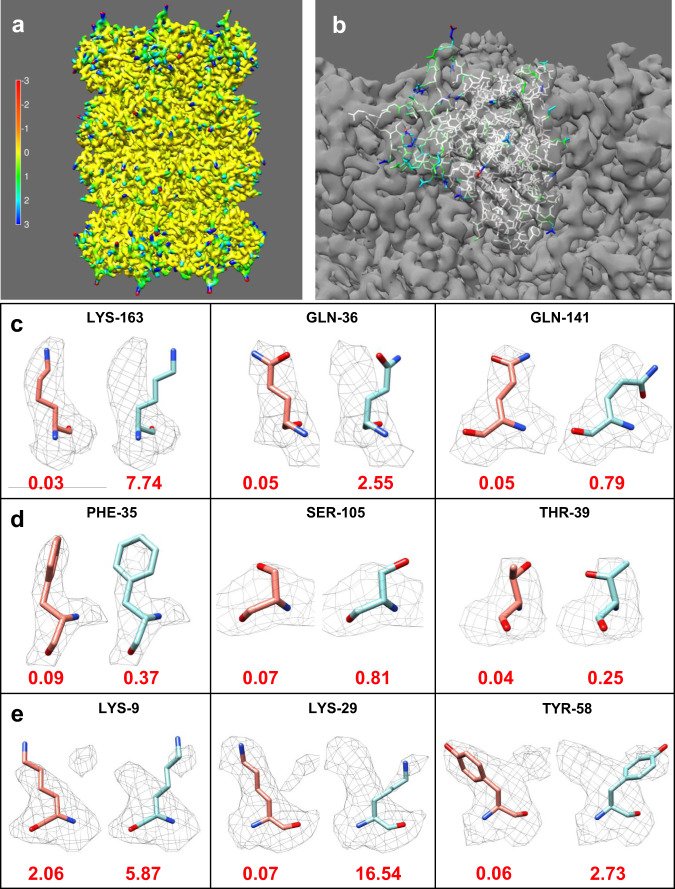
Detection of map-model fit errors using FSC-*Q*. FSC-*Q* values calculated for the 20 S proteasome structure (EMD-6287) are projected onto the map generated from the atomic model (pdb id: 6bdf) (**a**) or on the atoms of chain Q of the atomic model (**b**). Scale: red indicates atoms that may be associated with noise and blue indicates poorly fitted atoms or areas with low resolvability. **c**–**e** Rotamers of several amino acids in the 20 S proteasome structure that have been altered. In each panel, the original rotamer is shown on the left and the modified rotamer on the right with their corresponding average FSC-*Q*-scores. For the calculation of the average, the absolute FSC-*Q* value of each atom was considered. **c** Rotamers of three long-chain amino acids that are clearly out of the density (LYS-163 chain B, GLN-36 chain V, and GLN-141 chain T). **d** Very subtle modification of the residues: PHE-35 chain 1, SER-105 chain Q, and THR-39 chain R. **e** Residues in which the modified rotamers overlap with other densities (LYS-9 chain L, LYS-29 chain R, and THR-58 chain Z).

To test our algorithm, we initially used the known 20 S proteasome structure with a global resolution of 2.8 Å, as reported by the gold-standard FSC of 0.143 (EMD-6287)^12^ (Fig. 1), and the MD39 SOSIP trimer in complex with mature BG18 fragment antigen binding (EMD-7875)^13^ with an overall resolution of 4.4 Å (Supplementary Fig. 3). Figure 1 shows the FSC-*Q* values represented on a map generated from the 20 S proteasome atomic model (Fig. 1a) or on the atomic model itself (PDB: id-6bdf; Fig. 1b), while the Supplementary Fig. 3 shows FSC-*Q* and FSC-*Q*_R_ represented on the map generated with the MD39 SOSIP trimer model. Atoms with FSC-*Q* close to zero means that the model is supported by the signal in the half maps, while positive values far from zero corresponds to parts of the model that have a poor fit or that corresponds to areas in which the map had a poorer resolvability. On the other hand, negative values correspond to areas in which local resolution (calculated through FSC) between map and model is better than between the two halves, which typically corresponds to situations in which atoms are correlating with noise. In these specific cases, negative values correspond to external atoms of some side chains, where there is no signal in the map.

To test the usefulness of this measure, we artificially modified the side chain rotamers of some amino acids of the 20 S proteasome, using Coot^14^. Figure 1c–e shows the altered amino acids divided into three blocks. The first block (Fig. 1c) is made up of three long-chain amino acids (LYS and GLN) that are clearly out of the density. In the second block (Fig. 1d), there are very subtle modifications and short side chains errors, such as SER and GLN; finally, in the third block (Fig. 1e), we show cases in which the modified rotamers overlap with other densities. In all cases, the FSC-*Q* of the side chain increased compared to the original rotamer. A similar behavior is observed for FSC-Q_R_ in Supplementary Fig. 4. This shows that even subtle changes, such as aromatic ring rotation of the PHE-35 or side chain rotation of the SER-105 can indeed be detected by our method. We have also observed that although a relatively large sliding window is used for the calculation of the local resolution, the results are well localized. In all analyzed cases, we have seen that the artificial modification of the side chains is practically not affecting neighboring amino acids. A significant change was only detected in the case of LYS-29, where the artificial modification caused a clash with ASN-30 that was reflected in a change in FSC-*Q*. On the other hand, HIS-28 was not affected by the modification.

**Fig. 2:**
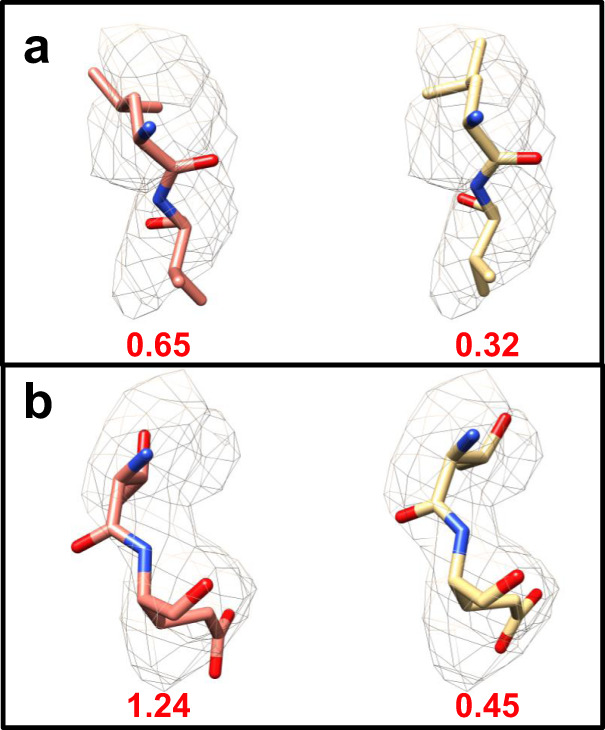
Improvement in the fit of the structure of the spike glycoprotein of SARS-CoV-2 in the closed state (EMD-21452). In each panel, the left side shows the original fit and the right side shows the improved fit, using ISOLDE^16^. **a** Fragment composed of amino acids LEU-533 and VAL-534, and **b** THR-323 and GLU-324. Under each fragment, the average FSC-*Q* is shown.

Furthermore, we analyzed the recent structure of the spike glycoprotein of SARS-CoV-2 in the closed state (EMD-21452), using half maps kindly provided by Prof. David Veesler^15^. Two refined models were obtained from the repository of Prof. Andrea Thorn at https://github.com/thorn-lab/coronavirus_structural_task_force, the one proposed by the authors and a model corrected, using ISOLDE^16^. As expected, the corrected model as a whole has a lower average FSC-*Q* (FSC-*Q* = 0.96 Å; FSC-*Q*_R_ = 0.29) compared to the original model (FSC-*Q* = 0.99 Å; FSC-*Q*_R_ = 0.30). These numbers may not seem too different, but it has to be taken into account that they refer to global averages. When analyzed locally, the number of atoms with FSC-*Q* > 0.5 Å drops by 615 (2.6% of the total atoms in the model) in the corrected model. Furthermore, our method identified some regions, such as amino acids LEU-533 to VAL-534 and THR-323 to GLU-324, where the corrected model clearly fits better to the experimental data than the original model (Fig. 2). In both areas, a considerable reduction of FSC-*Q* is observed.

### Overfitting

One of the big issues during the refinement of an atomic model is to detect and avoid overfitting. In the context of this work, overfitting occurs when the generated model is not supported by the reproducible signal on the half maps. Different strategies have been presented to detect it. For example, the calculation of the FSC between one half-map and a map generated from the model refined against another half-map^17^, or a comparison between the FSC of the original data and the FSC obtained, using data with noise introduced at high resolutions^18^. However, in both cases the problem is addressed globally.

**Fig. 3:**
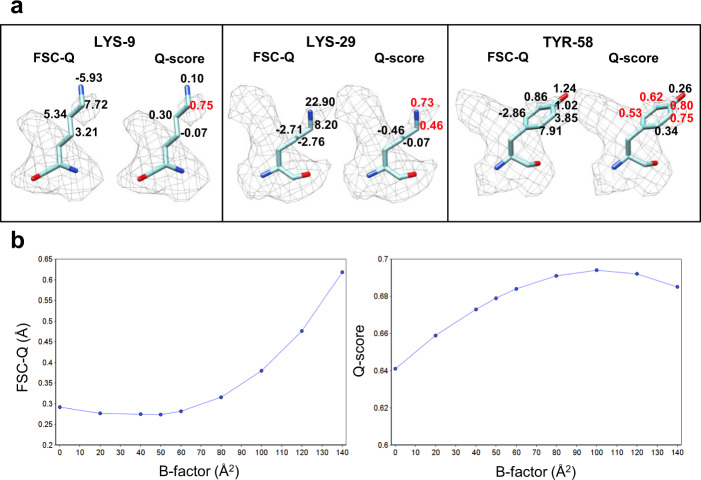
Comparison between FSC-*Q* and *Q*-score. **a** FSC-*Q* and *Q*-scores for each side chain atom for the same residues as in Fig. 1e. **b** The plots show the effect of *B*-factor sharpening on average FSC-*Q* and *Q*-scores analyzed using the 20 S proteasome map.

**Fig. 4:**
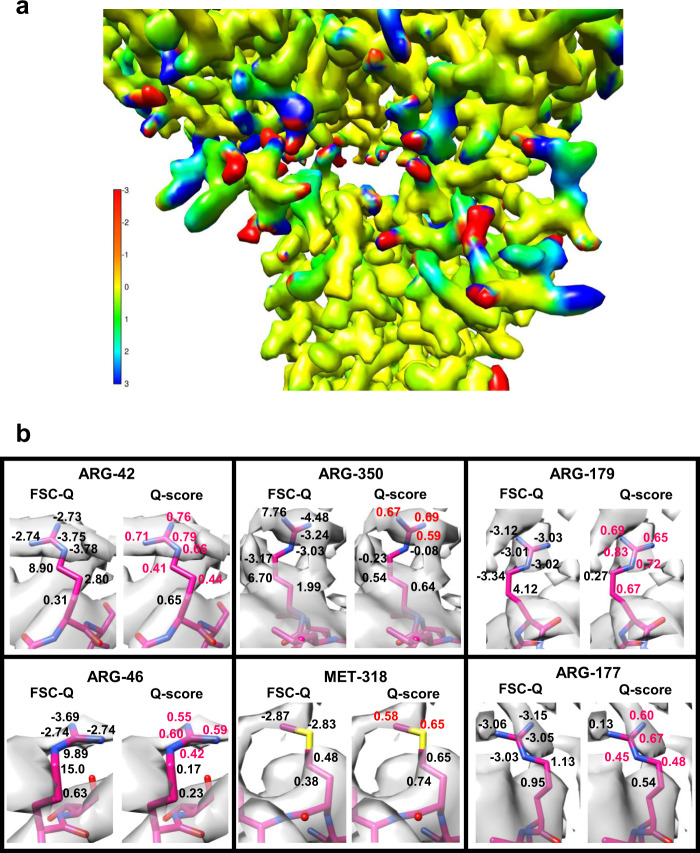
FSC-*Q* for a fragment of the PAC1 GPCR receptor complex (EMD-20278). **a** FSC-*Q* values superimposed on the map generated from the atomic model (pdb id: 6p9y). Red areas indicate side chains that fit into densities of the detergent micelle. Blue areas correspond to atoms with very low resolvability. **b** FSC-*Q* and *Q*-scores values are shown on each side chain atom of some residues that fit to the detergent (ARG-42 chain B, ARG-350 chain R, ARG-179 chain R, MET-318 chain R, GLU-58 chain G, and ARG-177 chain R).

The method we present here allows detecting the overfitting locally. Figures 1e and 3a show that when the side chains are adjusted to densities corresponding to refinement artifacts, the FSC-*Q* values largely diverge from 0. Another example is shown in Fig. 4, where the FSC-*Q* for PAC1 GPCR receptor complex (EMD-20278)^19^ has been represented. Figure 4a shows several areas in red, which coincide with side chains that fall on densities corresponding to the detergent micelle. Examples of some of these amino acids are shown in Fig. 4b. The particular case of ARG-42 is shown in Supplementary Fig. 5, and it can be seen that as the threshold is increased (Supplementary Fig. 5b) the densities belonging to the micelles cover the outer atoms of the side chain. In the amino acids studied (Fig. 4b), we observe that when the atoms fit to the detergent densities, FSC-*Q* moves away from zero and, importantly, the FSC-*Q* becomes negative.

### FSC-*Q* as a complement to *Q*-score

The *Q*-score method measures the correlation between the map values at points around the atom and a reference Gaussian-like function^10^. In this way, the *Q*-score estimates the resolvability of each atom with respect to the map from which the model has been refined. On the other hand, the method we present in this work measures how much of the model is supported by the signal in the half maps. In addition, we also include the environment of that atom within the local resolution window, spanning five times the global resolution. Although these methods measure different quantities, they both give a measure of the local quality-of-fit and complement each other.

The *Q*-score method is a good tool to estimate the resolvability of atoms. However, due to its normalization with respect to the mean, it is not capable of differentiating between the signal corresponding either to atoms or to densities generated by noise or artifacts of the reconstruction. Figure 3a shows the modified rotamers of the third block of Fig. 1e, with the FSC-*Q* and *Q*-score values for the atoms of the side chain. Several atoms are observed where the *Q*-score indicates a very good resolvability (in red color); however, these atoms are fitting into densities corresponding to noise. In contrast, the FSC-*Q* values for these atoms are very high, indicating a poor fit. This issue may have significant implications depending on the type of specimens. Particularly, for the very important biomedical case of membrane proteins, while FSC-*Q* is capable to identify the side chains where the fit is on the densities corresponding to the detergent micelle (Fig. 4), the *Q*-score is not, reporting values that would still show these fits as good (Fig. 4b).

Both the *Q*-score method and our own one depend on the *B*-factor sharpening that has been applied to the reconstructed map. We note that for FSC-*Q* this dependency is not obvious, since the FSC is a measure that is invariant with respect to a multiplicative factor. In other words, the FSC, without a mask, is not affected by *B*-factor-based sharpening, although this fact is normally not clearly expressed in the literature. However, when a map is masked in real space, its Fourier transform is convolved with the Fourier transform of the mask, increasing the correlation between Fourier coefficients in a neighborhood so that the FSC correspondingly increases. Therefore, in our method the *B*-factor sharpening dependency is due to the mask used in calculation of the local resolution. For simplicity, we restrict ourselves to those cases in which sharpening is done globally, rather than on more precise local methods (such as Ramírez-Aportela et al.^20^), that however, would complicate the presentation of these results significantly. To evaluate this dependence, the proteasome (EMD-6287) was sharpened using different *B*-factor values, in the same way as in Ramírez-Aportela et al.^5^. In that paper, we showed that a *B*-factor greater than −60 Å^2^ produced over-sharpening. The results obtained for the average value of *Q*-score and FSC-*Q* for each *B*-factor are graphically represented in Fig. 3b. The charts show that FSC-*Q* decreases slightly, reaching the minimum approximately for a *B*-factor of −50 Å^2^ (in this particular case), with a difference of 0.018 Å with respect to the unsharpened map. From that point on, it begins to grow abruptly with the increase in the *B*-factor. This result indicates that FSC-*Q* does not favor over-sharpened maps. On the other hand, the charts show an increase in the *Q*-score, which reaches the maximum for a *B*-factor of −100 Å^2^, with a difference of 0.053 with respect to the unsharpened map. From there the *Q*-score decreases slightly. Taken together, this analysis suggests that *Q*-score rewards over-sharpening, and this effect may be a consequence of the normalization used, while FSC-*Q* does not. We note that this analysis has been done for global *B*-factor-based sharpening, but it can be extrapolated to local sharpening methods as well.

### FSC-*Q* versus resolution and ADPs

To study the behavior of FSC-*Q* and FSC-*Q*_R_ with respect to resolution, a set of 50 maps and models obtained from EMDB were analyzed with resolutions between 1.25 and 5 Å (Supplementary Fig. 6). The data set is composed of maps and models published between November and December 2019, with resolutions better than 5 Å and for which half maps have been deposited (39 cases). In addition, the nine structures presented in the Supplementary Table 1 of Pintilie et al.^10^, in which half maps are deposited have also been taken into account. Finally, the two recently deposited structures with higher resolution are included (EMD-11103 and EMD-11122). For this data set, the average absolute FSC-*Q* and FSC-*Q*_R_ for the entire map were calculated and plotted as a function of the reported resolution.

As can be seen in Supplementary Fig. 6, as the resolution decreases, FSC-*Q* and FSC-*Q*_R_ increase. However, we can note that in most cases for resolutions better than 3 Å, the values of FSC-*Q* and FSC-*Q*_R_ are lower than 0.5 and 0.2 Å, respectively. For resolutions between 3 and 4 Å both metrics grow up to 1.5 Å for FSC-*Q* and 0.30 for FSC-*Q*_R_. It is known that the complexity to build an atomic model increases at resolutions lower than 3 Å and many side chains are not visible at 3.5 Å. In almost all cases for resolutions worse than 4 Å, we found an increase of FSC-*Q* (FSC-*Q* > 1.5 Å and FSC-*Q*_R_ > 0.3). Note that for resolutions lower than 4 Å, model building is a challenge (e.g., beta-strands become visible at resolutions ~4.5 Å).

We also analyzed the information relationship between global resolution and (for simplicity) volume-averaged values of FSC-*Q*, FSC-*Q*_R_, and *Q*-score. A principal component analysis (PCA) of the correlation matrix of the variables is shown in Supplementary Fig. 7. It is clear that there is a linear relationship between resolution and *Q*-score, and a nonlinear one between FSC-*Q* and resolution. The linearity between FSC-*Q* and FSC-*Q*_R_ shows that they are simply two different ways of looking at the same source of information (as expected). The first principal component of the analysis explained 81% of the observed variability. This is an indication that all measurements (overall resolution, FSC-*Q*, FSC-*Q*_R_, and *Q*-Score) are mostly related to a single source of information of the map, namely, its quality in terms of resolution. The first two principal components explain 98% of the variability and, very importantly, the information of the second principal component comes mostly from FSC-*Q* (this is shown in the biplot of Supplementary Fig. 7b), meaning that this measure is bringing a new source of information that was not available before (even after considering *Q*-score), in particular, the quality of the fitting between the map and the model, especially for low resolution (>3 Å) maps. Another way to understand the novelty brought by this measure is to realize that *Q*-score and resolution lie on almost the same axis in the biplot (i.e., they bring similar information), while FSC-*Q* and FSC-*Q*_R_ lie in an almost orthogonal direction (i.e., they bring information that is not aligned with previous knowledge). Despite its novelty (being orthogonal to resolution), the informational content of the data set is dominated by resolution, with the first eigenvalue of PCA explaining 81% of the variance. Understood in this way, FSC-*Q* is adding new ways to compare differences among the CryoEM map and the atomic model. Note also that FSC-*Q* has a small slope for maps whose resolution is better than 3 Å, indicating that for these maps the tracing of the model is a relatively safe process, while it shows a high variability for maps whose resolution is worse than 3 Å, meaning that for these maps the correct tracing of the map is much harder.

We further analyzed the relationship of FSC-*Q* and FSC-*Q*_R_ to atomic displacement parameters (ADPs, also known as *B*-factors). In Supplementary Fig. 8, we present a detailed analysis of EMD-20259 map at 3.57 Å reported resolution. The figure shows a weak correlation of FSC-*Q* and FSC-*Q*_R_ with ADPs, indicating that both measures convey different information. While ADPs give a measure of the positional uncertainty of the atom based largely on geometric constraints, FSC-*Q* focuses on how good the fit is in relation to the reproducible signal in the half maps. From Supplementary Fig. 8, it is interesting to see that when the ADP is low (there is little uncertainty relative to geometric model constraints), the FSC-*Q* is also low (its position is consistent with the data).

## Discussion

The ultimate goal of most single-particle cryoEM experiments is to generate an atomic model from the refined density map. However, the resolution of cryoEM maps may change in different areas and building an accurate atomic model, using the map can be a real challenge. The development of metrics for map-to-model fit validation is a critical step towards robust generating high accuracy models from cryoEM maps.

In this paper, we proposed an approach to analyze the fit of the models to the density maps. Our method is a significant extension of the map-to-model FSC calculation that is commonly used to assess goodness-of-fit^21^, since it explicitly introduces locality. Here, we calculate the map-to-model local resolution map (based on FSCs) and compare it with the half maps local resolution. Differences between them allow us to select an objective signal threshold so that we can identify those areas, where the model disagrees with the consistent signal on the half maps.

We have shown that FSC-*Q* enables identification of errors in fit and local detection of overfitting. Overfitting is one of the main challenges during model refinement and new metrics to detect it are needed^22^. The *Q*-score has been indicated by the authors^10^ that is particularly sensitive to overfitting. Furthermore, we have shown that our method has less dependence on sharpening than the map *Q*-score, and that they complement each other to better estimate the resolvability of atoms. Indeed, the combination of these (and other) methods probably will lead to meta validation measurements, where the combination of methods as “orthogonal” as possible will lead to new simple metrics, yet to be explored, which could be directly incorporated into modeling workflows.

## Methods

### Local quality of fit

A common practice to globally assess the quality of the fit between the reconstructed map and the refined model is the use of global FSC. However, this practice only allows the fitting to be assessed as a whole. Here, we implement an algorithm to evaluate the quality of the fitting of the atomic model into the density map following a similar strategy, but locally.

Our algorithm follows the general principle of making a comparison between the full data set and the map generated from the atomic model, now using the local resolution map (${{V}}_{{\mathrm{FSC}}_{{\mathrm{map}} - {\mathrm{model}}}}$; note that the values of this volume are measured in Å). However, the key novelty is that we then compare it with the local resolution map obtained when using the half maps (${{V}}_{{\mathrm{FSC}}_{{\mathrm{half}}}}$). We define FSC-*Q* as the difference map between ${{V}}_{{\mathrm{FSC}}_{{\mathrm{map}} - {\mathrm{model}}}}$ and ${{V}}_{{\mathrm{FSC}}_{{\mathrm{half}}}}$.

1 $${\mathrm{FSC}}-{{Q}} = {{V}}_{{\mathrm{FSC}}_{{\mathrm{map}} - {\mathrm{model}}}} - {{V}}_{{\mathrm{FSC}}_{{\mathrm{half}}}}.$$

Note that this map has values that encode the differences in Å. Obviously, this combination of local resolution maps calculated from half the data set and from the full data set requires an appropriate statistical analysis, which will be addressed in the next section. For the calculation indicated in Eq. (1), a sliding window of size five times the overall resolution reported is used to calculate the local resolution maps, following the analysis in Cardone et al.^2^. A soft mask is applied to enclose the region of interest of the macromolecule. Those points of the map where there are “significant” differences between ${{V}}_{{\mathrm{FSC}}_{{\mathrm{map}} - {\mathrm{model}}}}$ and ${{V}}_{{\mathrm{FSC}}_{{\mathrm{half}}}}$, allow us to detect errors in the refinement of the model or uncertainties in positions of parts of the model.

### FSC threshold

Equation 1 compares a resolution map determined from half the data set with a resolution map obtained from the complete data set. Certainly, this is not a simple issue and in the following we present how we perform this task using a rather simple and, we think, uncontroversial and widely accepted reasoning. We recall that Cardone et al.^2^, proposed a FSC threshold of 1/2 to report the local resolution. We will start by choosing this threshold for the analysis of the FSC between two half maps. For the comparison of the map obtained with the full data set and the map obtained from the atomic model, we start by acknowledging that we do not have a proper model for the statistical distribution of the differences, since there are many ways in which an atomic model may deviate from the unknown “truth”. For this reason, our simplifying hypothesis is that the error distribution in the map derived from the model follows the same distribution as a map obtained from a full data set, we would then be comparing two independent maps from two full sets of images, using as a threshold of 2/3, the one proposed for this case by Rosenthal and Henderson^23^. We therefore do not claim FSC-*Q* to be a measure of resolution discrepancy, but a useful tool to identify regions that are not well supported by the signal content obtained in the two half maps. In addition, we experimentally show that the distribution of FSC-*Q* values fluctuates ~0 when the map and model match (Supplementary Fig. 1), which reflects the unbiased nature of the FSC-*Q* measure when these two thresholds (1/2 and 2/3) are used.

### Input and output

The algorithm requires as input a 3D cryoEM density map, the fitted atomic model, the two half maps, and a soft mask enclosing the macromolecule. The blocres program^2^ is used to calculate the ${{V}}_{{\mathrm{FSC}}_{{\mathrm{map}} - {\mathrm{model}}}}$ and ${{V}}_{{\mathrm{FSC}}_{{\mathrm{half}}}}$. To assess the quality of the model fit, the ${{V}}_{{\mathrm{FSC}}_{{\mathrm{half}}}}$ map is subtracted from the ${{V}}_{{\mathrm{FSC}}_{{\mathrm{map}} - {\mathrm{model}}}}$ map (Eq. 1), generating a difference map with the values encoded in Å. We find it useful to project this difference map onto atomic models (PDB format). We created a specific tool xmipp_pdb_label_from_volume, in XMIPP3 (ref. ^24^), which represents the map values in the occupancy column for each atom in the model.

In addition, since the value of FSC-*Q* relating to resolution may be useful (i.e., a difference of 0.5 Å is not the same in a region with resolution of 2 Å, as it is in a region with resolution of 4 Å), the term FSC-*Q*_R_ has been introduced by dividing FSC-*Q* by the local resolution map as follows:

2 $${\mathrm{FSC}}-{{Q}}_{\mathrm{R}} = ({{V}}_{{\mathrm{FSC}}_{{\mathrm{map}} - {\mathrm{model}}}} - {{V}}_{{\mathrm{FSC}}_{{\mathrm{half}}}})/{{V}}_{{\mathrm{FSC}}_{{\mathrm{half}}}}.$$

While FSC-*Q* gives a measure of the difference between the local resolution of the half maps and map-model due to model fit, FSC-*Q*_R_ explains the fraction of the local resolution that the disagreement between the local resolution of the two half maps and the final map and the atomic model represents. For example, FSC-*Q*_R_ = 0.3 means that the disagreement between the two local resolutions (${{V}}_{{\mathrm{FSC}}_{{\mathrm{map}} - {\mathrm{model}}}}$ and ${{V}}_{{\mathrm{FSC}}_{{\mathrm{half}}}}$) represent 30% of the local resolution (${{V}}_{{\mathrm{FSC}}_{{\mathrm{half}}}}$).

In all the cases analyzed in this work, the function xmipp_volume_from_pdb^25^ was used to generate the density map from the atomic model, using the atomic form factors for electron scattering. From this map, a soft mask was generated using the xmipp–create 3d mask protocol in *Scipion*^26^. A threshold of 0.02 and a dilation of 3 pixels were applied. In all cases, a sliding window of five times the reported resolution was used, except for maps with resolution better than 2 Å, where the window size was kept at 11 pixels/voxels to prevent instabilities in the calculation of FSC-*Q* (see Supplementary Fig. 2, where an instability analysis for very high-resolution maps is presented).

FSC-*Q* has been integrated into *Scipion3* (ref. ^26^). Different visualization options have been implemented to analyze the results. For example, the display of the map or the atomic model in UCSF Chimera^27^ colored according to the calculated FSC-*Q* or FSC-*Q*_R_ values. In addition, the number of atoms (and percent) with FSC-*Q* > 0.5 Å or FSC-*Q* < −0.5 Å is determined and the amino acids containing the poor fitted atoms (FSC-*Q* > 0.5 Å or FSC-*Q* < −0.5 Å) are shown in tables.

## Supplementary information

Supplementary Information

## Data Availability

All published data sets used in this paper were taken from the EMDB and PDB (accession codes specified in the figure captions, see also the table in Supplementary Fig. 6). Other data are available from the corresponding authors upon reasonable request.

## Code availability

The protocol used here has been integrated into the image-processing framework *Scipion*^26^, in the development branch https://github.com/I2PC/scipion-em-xmipp (this branch will eventually become the next release of *Scipion3*). The name of the protocol is *validate fsc-q*.
