# Supplementary material for: FSC-Q: a CryoEM map-to-atomic model quality validation based on the local Fourier shell correlation
Source: Nat Commun. 2021 Jan 4;12:42. doi: 10.1038/s41467-020-20295-w (PMC7782520; doi:10.1038/s41467-020-20295-w)
Supplement: Supplementary file 1 — Supplementary Information [file 41467_2020_20295_MOESM1_ESM.pdf]

# Supplementary Information

# FSC-Q: A CryoEM map-to-atomic model quality validation based on the local Fourier Shell Correlation

**Erney Ramírez-Aportela**[*1], **David Maluenda**[1], **Yunior C. Fonseca**[1], **Pablo Conesa**[1], **Roberto Marabini**[2], **J. Bernard Heymann**[3], **Jose Maria Carazo**[*1], **Carlos Oscar S. Sorzano**[*1,4]

[1]Biocomputing Unit, National Center for Biotechnology (CSIC), Darwin 3, Campus Univ. Autónoma de Madrid, 28049 Cantoblanco, Madrid, Spain.

[2]Univ. Autónoma de Madrid, Campus Univ. Autónoma de Madrid, 28049 Cantoblanco, Madrid, Spain.

[3] Laboratory of Structural Biology Research, NIAMS, NIH, Bethesda, MD, USA.

[4]Univ. CEU San Pablo, Campus Urb. Montepríncipe, Boadilla del Monte, 28668 Madrid, Spain.

*Corresponding authors: erney.ramirez@gmail.com, carazo@cnb.csic.es, coss@cnb.csic.es

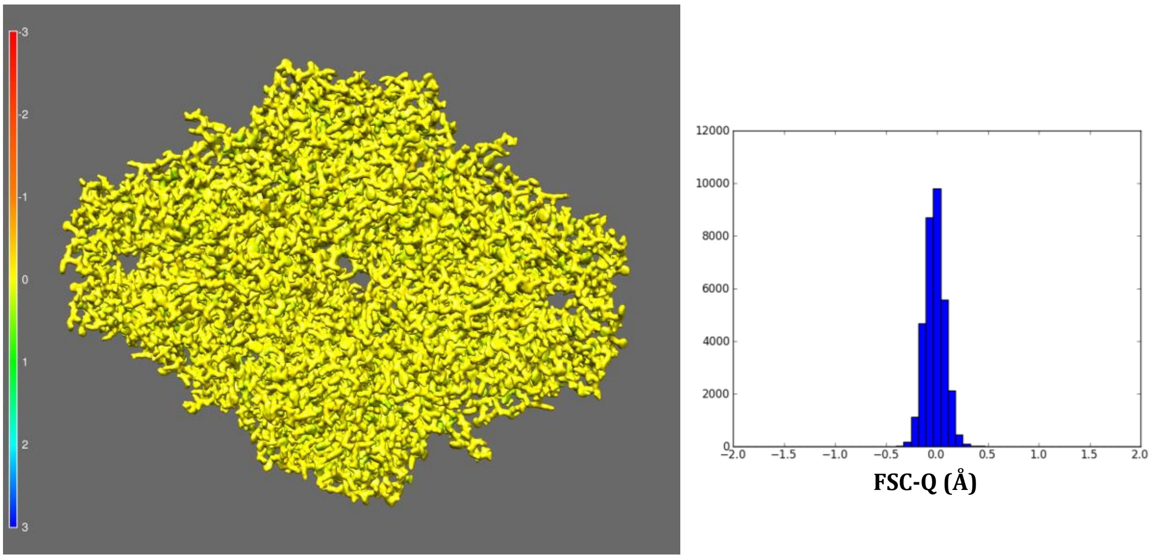


**Supplementary Fig. 1. FSC-Q distribution for optimal map-to-model fit.** For optimal fit, a map was reconstructed from the ß-galactosidase atomic model (pdb id: 3j7h), carrying out the following protocol on *Scipion*. First, a map was generated from the atomic model. Using the map, projections were generated in all directions with an angular sampling of 1.5 degrees, for a total of 18,309 projections. Gaussian noise with zero mean and a standard deviation of 50 was added to the set of projections and map reconstruction was carried out using the *RELION* software [1].

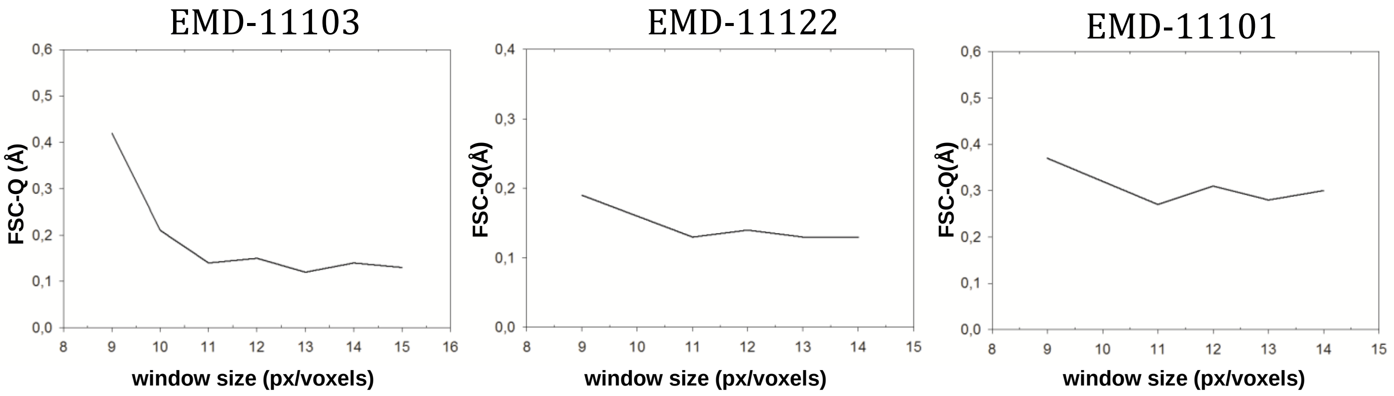


**Supplementary Fig. 2. Effect of using different window sizes on the FSC-Q for high resolution maps.** The effect of the window size on the FSC-Q is studied for three high resolution maps, EMD-11103, EMD-11122 and EMD-10101 with resolutions of 1.25 Å, 1.56 Å and 1.84 Å respectively. The plots show that from a window size of 11 px/voxels the FSC-Q is more stable.

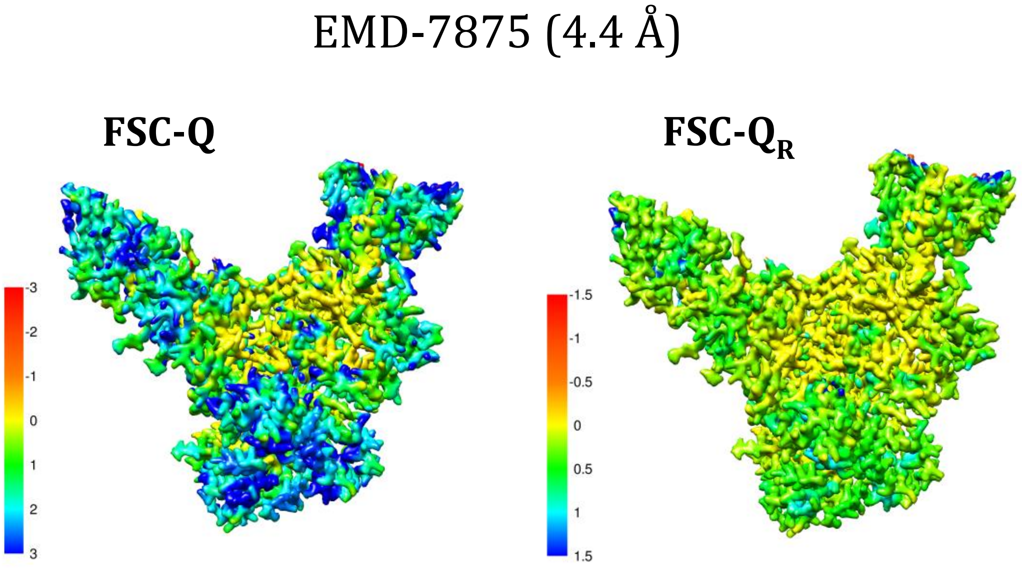


**Supplementary Fig. 3. FSC-Q and FSC-$Q_R$ values calculated for the MD39 SOSIP trimer structure (EMD-7875).** The values are represented on the map generated from the atomic model (pdb id: 6dfg).

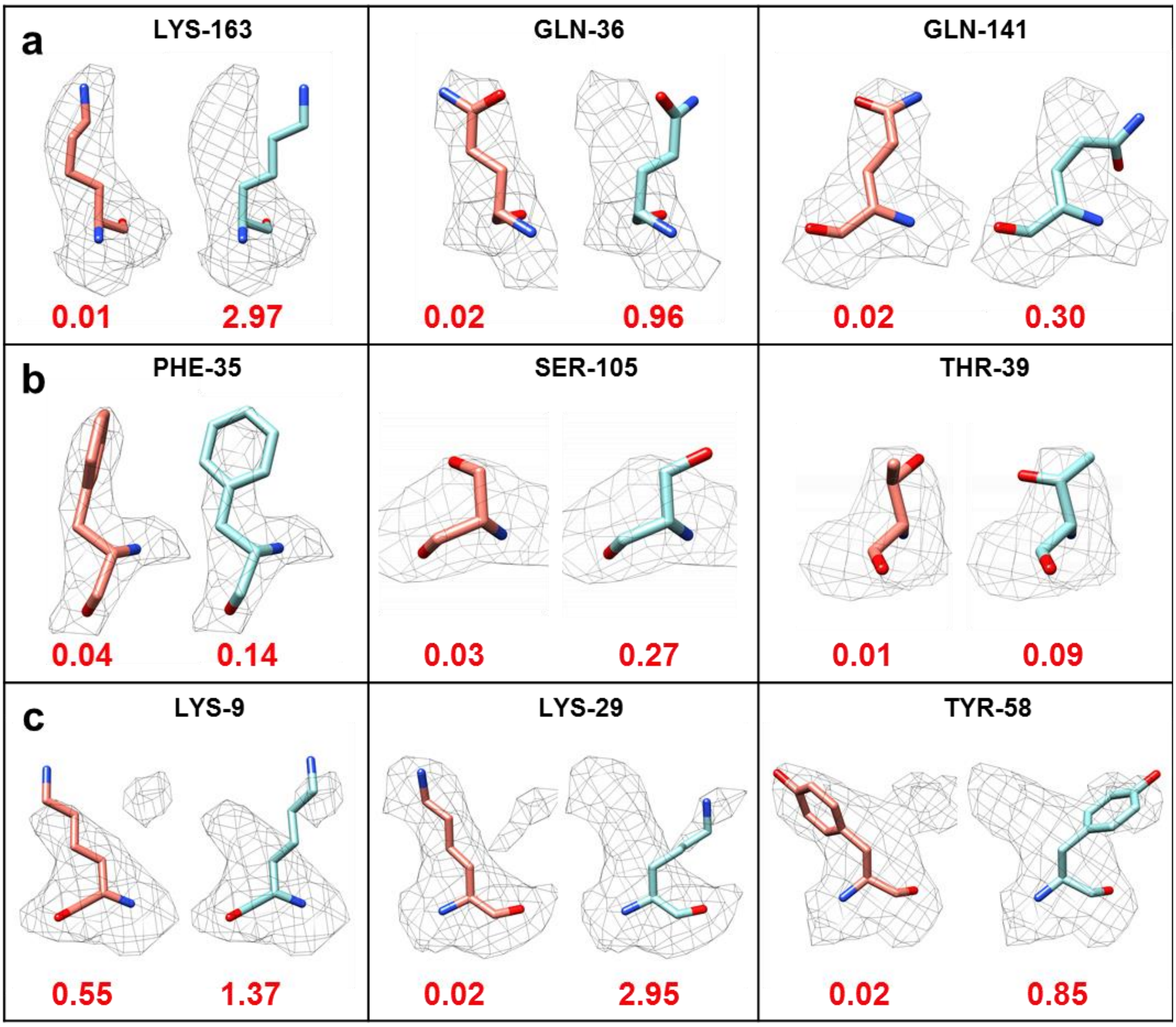


**Supplementary Fig. 4. Detection of map-model fit errors using FSC-$Q_R$.** The panels show rotamers of several amino acids in the 20S proteasome structure that have been altered. In each panel, the original rotamer is shown on the left and the modified rotamer on the right with their corresponding average FSC-$Q_R$ scores. For the calculation of the average, the absolute FSC-$Q_R$ value of each atom was considered. **a** Rotamers of 3 long-chain amino acids that are clearly out of the density [LYS-163 chain B, GLN-36 chain V and GLN-141 chain T]. **b** Very subtle modification of the residues: PHE-35 chain 1, SER-105 chain Q and THR-39 chain R. **c** Residues in which the modified rotamers overlap with other densities [LYS-9 chain L, LYS-29 chain R and THR-58 chain Z].

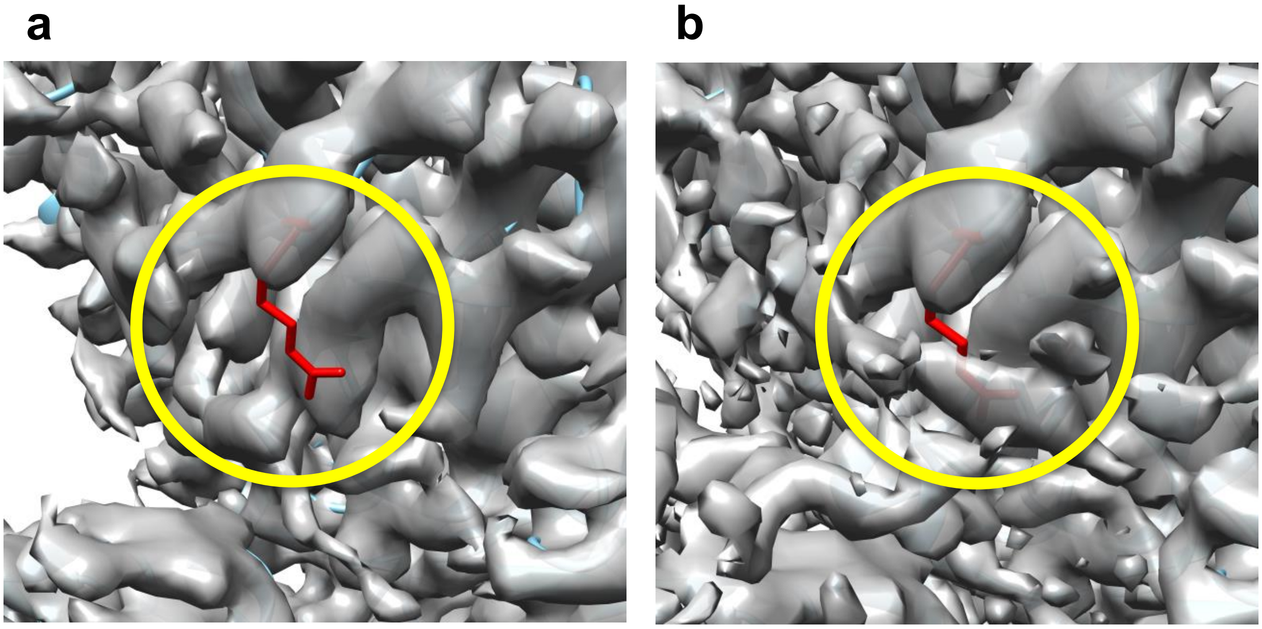

**Supplementary Fig. 5. Micelle identification for PAC1 GPCR Receptor complex (EMD-20278). a** Zoom on the residue ARG-42 chain B (shown in red). **b** As the threshold increases, the micelle densities overlap with the side chain of ARG-42.

**a**

| EMD ID | Resolution (Å) | FSC-Q (Å) | FSC-$Q_R$ | Q-score |
|---|---|---|---|---|
| 11103** | 1,25 | 0,14 | 0,13 | 0,94 |
| 11122** | 1,56 | 0,13 | 0,09 | 0,9 |
| 10101* | 1,84 | 0,27 | 0,17 | 0,9 |
| 7770* | 1,9 | 0,19 | 0,1 | 0,71 |
| 4905* | 2,1 | 0,25 | 0,12 | 0,83 |
| 2984* | 2,2 | 0,34 | 0,13 | 0,62 |
| 8908* | 2,2 | 0,31 | 0,13 | 0,69 |
| 20839 | 2,7 | 0,29 | 0,11 | 0,68 |
| 10181 | 2,8 | 0,39 | 0,09 | 0,6 |
| 10468 | 2,8 | 0,34 | 0,1 | 0,58 |
| 4888 | 2,8 | 0,41 | 0,12 | 0,61 |
| 4889 | 2,9 | 0,27 | 0,07 | 0,65 |
| 10418 | 2,96 | 0,31 | 0,09 | 0,61 |
| 20538 | 3 | 0,12 | 0,04 | 0,65 |
| 20846 | 3,04 | 0,48 | 0,14 | 0,59 |
| 20594 | 3,05 | 0,2 | 0,06 | 0,63 |
| 10420 | 3,1 | 0,43 | 0,11 | 0,56 |
| 10380 | 3,1 | 0,49 | 0,1 | 0,53 |
| 4890 | 3,1 | 0,28 | 0,07 | 0,49 |
| 20668 | 3,1 | 0,79 | 0,18 | 0,54 |
| 20540 | 3,17 | 0,36 | 0,13 | 0,64 |
| 10069 | 3,2 | 0,35 | 0,08 | 0,53 |
| 10333 | 3,2 | 0,26 | 0,07 | 0,61 |
| 4907 | 3,2 | 0,34 | 0,11 | 0,56 |
| 5995* | 3,2 | 0,62 | 0,18 | 0,54 |
| 4595 | 3,28 | 0,78 | 0,25 | 0,49 |
| 10049 | 3,3 | 0,76 | 0,24 | 0,52 |
| 20806 | 3,3 | 0,43 | 0,13 | 0,57 |
| 5623* | 3,3 | 0,4 | 0,12 | 0,6 |
| 10312 | 3,4 | 1,15 | 0,46 | 0,46 |
| 10419 | 3,4 | 0,38 | 0,1 | 0,52 |
| 7885 | 3,4 | 0,44 | 0,11 | 0,53 |
| 20847 | 3,54 | 0,51 | 0,11 | 0,48 |
| 9187 | 3,54 | 0,89 | 0,2 | 0,36 |
| 20259 | 3,57 | 0,7 | 0,19 | 0,49 |
| 20541 | 3,6 | 0,31 | 0,07 | 0,5 |
| 7884 | 3,7 | 0,93 | 0,22 | 0,46 |
| 2764* | 3,75 | 0,72 | 0,12 | 0,42 |
| 9188 | 3,84 | 1,29 | 0,27 | 0,29 |
| 7876 | 3,85 | 0,77 | 0,18 | 0,44 |
| 20260 | 3,94 | 1,18 | 0,26 | 0,42 |
| 10290 | 4,2 | 4,33 | 0,76 | 0,28 |
| 0193 | 4,3 | 3,58 | 0,61 | 0,31 |
| 10273 | 4,3 | 2,69 | 0,55 | 0,29 |
| 10467 | 4,32 | 1,98 | 0,32 | 0,15 |
| 4980 | 4,4 | 1,87 | 0,34 | 0,29 |
| 7875 | 4,42 | 1,39 | 0,28 | 0,33 |
| 2677* | 4,5 | 2,26 | 0,37 | 0,28 |
| 10294 | 4,6 | 3,2 | 0,52 | 0,23 |
| 20840 | 4,9 | 0,86 | 0,12 | 0,23 |

**b**

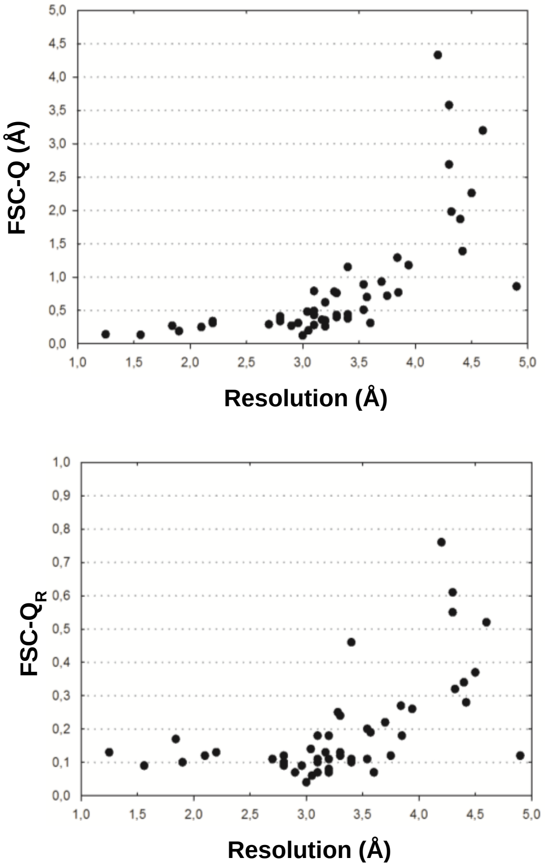


**Supplementary Fig. 6. Set of maps from EMDB for which overall absolute FSC-Q, FSC-$Q_R$ and Q-score were calculated.** **a** A set of 50 maps are used for the calculation. The set consists of 39 maps published between November and December 2019, 9 maps (marked with *) selected from Supplementary Table 1 of Pintilie et al., 2020, and the two highest resolution maps published recently (marked with **). **b** Average absolute FSC-Q and FSC-$Q_R$ versus reported resolution.

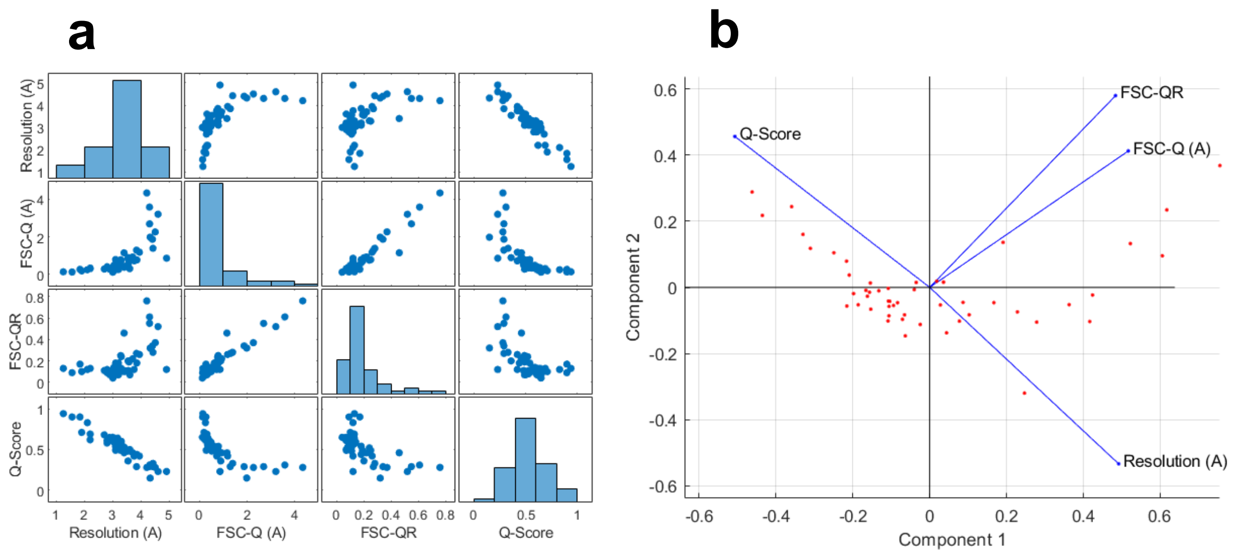


**Supplementary Fig. 7. Principal Component Analysis of the correlation matrix of FSC-Q, FSC-Q$_R$ and Q-score for the cases shown in Supplementary Fig. 6. a** Scatter plots and histograms of the variables being compared. **b** Biplot of the Principal Component Analysis of the four variables being compared (Resolution, FSC-Q, FSC-Q$_R$, and Q-Score).

EMD-20259

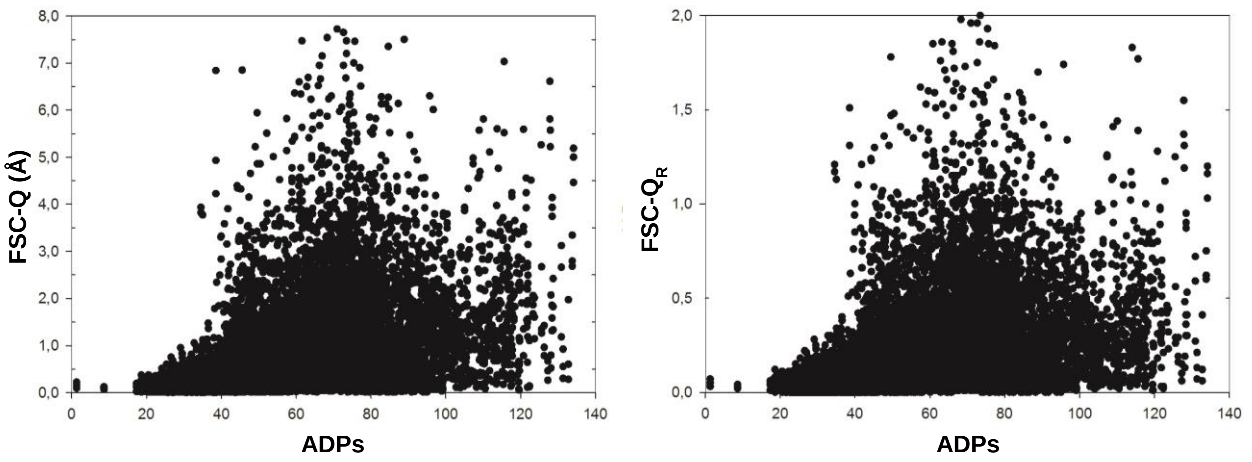


**Supplementary Fig. 8. Correlation between atomic displacement parameters (ADPs) and FSC-Q (left) or FSC-$Q_R$ (right), calculated on the EMD-20259 map with global resolution of 3.57 Å.** Each point represents an atom in the atomic model. Note that the absolute values of FSC-Q and FSC-$Q_R$ are represented.

## References


1. Scheres, S.H. RELION: implementation of a Bayesian approach to cryo-EM structure determination. J Struct Biol 180, 519-30 (2012).
